# Insights of red cell distribution width for mortality in septic patients with diabetes mellitus: A multicenter cohort study

**DOI:** 10.1371/journal.pone.0333689

**Published:** 2025-10-07

**Authors:** Kunsheng Zhao, Guang Zhang, Chengsheng Wu, Dawei Wang, Hongyu Zhang

**Affiliations:** 1 Department of Health Management, The First Affiliated Hospital of Shandong First Medical University & Shandong Provincial Qianfoshan Hospital; Shandong Engineering Research Center of Health Management; Shandong Institute of Health Management, Jinan, Shandong, China; 2 Department of Infectious Diseases, The Second Affiliated Hospital of Shandong First Medical University, Taian, Shandong, China; University of Cape Town Faculty of Science, SOUTH AFRICA

## Abstract

The correlation between red cell distribution width (RDW)and mortality in septic patients with diabetes mellitus has not been extensively investigated. This study aimed to explore the correlation between RDW and mortality and potential value of RDW as a prognostic indicator in septic patients with diabetes mellitus. A total of 5476 septic patients with diabetes mellitus were included in this multicenter retrospective cohort study. Multivariate logistic regression, dose-response, and mediation analyses were conducted to examine the association between RDW and mortality in septic patients with diabetes mellitus. The predictive value of RDW was assessed using the receiver operating characteristic (ROC) curve analysis and SHapley Additive exPlanations (SHAP) analysis. The improvement in the model was assessed using net reclassification improvement (NRI) and integrated discrimination improvement (IDI). After adjusting for all confounders, RDW was an independent variable for mortality (odds ratio [OR]: 1.37, 95% confidence interval [CI]: 1.26, 1.50, per standard deviation in RDW, *P* < 0.001). Moreover, RDW was positively correlated with 28-day mortality in a non-linear manner. The contribution of the predictive value of RDW was substantial in the ROC curve and SHAP analyses. The addition of RDW improved the predictive performance of the baseline model (continuous NRI [95% CI], 0.284 (0.188, 0.379); *P* < 0.001; IDI [95% CI], 0.012 (0.007, 0.016); *P* < 0.001). In conclusion, RDW may be a valuable indicator for predicting risk stratification and outcomes in septic patients with diabetes mellitus.

## Introduction

Sepsis is a common and lethal syndrome that causes rapid deterioration, is accompanied by multiple organ dysfunction, and remains one of the leading causes of death worldwide [1,2]. In 2017, there were approximately 50 million cases of sepsis worldwide, nearly one-quarter of which resulted in death, accounting for nearly one-fifth of all deaths worldwide [3]. Sepsis is the disease with the highest hospital treatment costs in the United States, with over 24 billion dollars spent on total hospital expenses annually [3]. In recent years, the results of various clinical trials for sepsis treatment have been unsatisfactory, with repeated failures reported [4–7]. No significant changes in mortality were observed from 2002 to 2016 after adjusting for disease severity [8]. Therefore, the early identification of risk factors and timely intervention may be particularly important to improve treatment and reduce costs. The application of several effective indicators and scoring systems is limited by factors such as detection level, operational complexity, and cost [9–11]. Therefore, a simple and reliable method for predicting prognosis in septic patients is urgently required.

The red cell distribution width (RDW), calculated based on the mean corpuscular volume of erythrocytes, is an indicator of the heterogeneity of erythrocyte size [12]. An increase in RDW suggests that some erythrocytes are either too large or too small, which may be indicative of several abnormalities such as oxidative stress, hypertension, poor nutritional status, inflammation, erythrocyte fragmentation, dyslipidemia, and alteration of erythropoietin function [12]. RDW is associated with several adverse outcomes such as acute heart failure [13], acute renal failure [14], pulmonary embolism [15], stroke [16], and peripheral artery disease [17]. Currently, RDW is widely available in laboratory testing, and its clinical value is increasing.

Several researchers have explored the relationship between RDW and mortality in patients [18–20]. However, most of these studies are small sample size studies on infants and young children. Furthermore, the relationship between septic patients with diabetes mellitus has not been extensively investigated. Diabetes mellitus is a complex disease that causes a series of complications and can exacerbate the progression of other conditions, resulting in a poor prognosis. Therefore, this relationship seems to be more complex in people with diabetes. Thus, this study aimed to explore the relationship and predictive value of RDW for mortality in septic adults with diabetes mellitus, using a large multicenter cohort.

## Methods

### Study population

All patients were enrolled from the eICU Collaborative Research Database (eICU-CRD). The database is a multicenter platform that includes data from 335 ICUs across 208 hospitals in the United States, collected between 2014 and 2015 [21,22]. The study was approved by the Institutional Review Board (IRB) of the Massachusetts Institute of Technology (MIT). Informed consent was waived for the retrospective design, lack of direct patient intervention. The request for additional ethical approval was also waived for this study by the IRB of MIT (record ID: 40859994). This study was conducted in accordance with the principles of the Declaration of Helsinki. Diagnosis of eICU database was based on the International Classifcation of Diseases, 9th Edition, Clinical Modifcation (ICD-9) codes. Patients with a primary diagnosis of sepsis, recorded on patient dataset (code: A41.9), were potentially eligible. The primary outcome was death in the ICU at 28 and 14 days.

A total of 5476 septic patients with diabetes mellitus on admission to the ICU were included according to the following exclusion criteria: (1) non-diabetes mellitus, (2) age < 18 years, (3) missing RDW, and (4) missing ICU outcomes. A flowchart of the study is presented in S1 Fig in S1 File.

### Statistical analysis

Continuous variables with a normal distribution were expressed as means ± standard deviation (SD), whereas continuous variables with a skewed distribution were expressed as the median and interquartile ranges (IQR). Categorical variables are expressed as numbers (%). Differences in continuous variables with a normal distribution were evaluated by the one-way analysis of variance (ANOVA). If continuous variables showed a distribution, the Kruskal-Wallis rank sum test was used to assess the differences. Chi-square tests were used to identify the differences in categorical variables. Kaplan-Meier survival curve analysis was conducted to test the difference in survival based on RDW tertiles. Univariate logistic regression models were used to identify related variables for mortality. Multivariate logistic regression models were used to examine the independent correlation between RDW and 28-day ICU mortality. Age, sex, and ethnicity were adjusted in Model 1. Variables with *P* < 0.05 in the univariate logistic analysis were adjusted in Model 2. Model 3 was adjusted for all variables in the univariate logistic analysis, including age, sex, ethnicity, chronic obstructive pulmonary disease (COPD), congestive heart failure (CHF), acute myocardial infarction (AMI), pneumonia, arrhythmias, body mass index (BMI), calcium, glucose, serum potassium, sodium, platelets, serum creatinine (Scr), white blood cells (WBC), and blood urea nitrogen (BUN). Multicollinearity was tested using the variance inflation factor (VIF). In this study, the VIFs for all variables were < 5. The dose-response analysis was estimated using a restricted cubic spline (RCS). A log-likelihood ratio test was performed to examine whether the dose-response relationship was nonlinear. If a non-linear relationship was found, a threshold analysis was performed based on the generalized additive model to explore the trend changes of the curve. Subgroup and interaction analyses were conducted across various variables. Mediation analysis was applied to test whether BMI mediated the relationship between RDW and mortality based on the product-of-coefficients method. Receiver operating characteristic (ROC) curve analysis was performed to evaluate the model’s improvement ability with and without RDW. The contribution of the predictive value of RDW was explored by using SHapley Additive exPlanations (SHAP) analysis based on the XGBoost model. Net reclassification improvement (NRI) and integrated discrimination improvement (IDI) were performed to further evaluate the model discrimination. The cutoff points for classification NRI are set at 0.2 and 0.4. A sensitivity analysis was performed based on the 14-day mortality outcome to verify the stability of the results. Additionally, E-values were calculated to evaluate the effects of potentially unmeasured confounders. PASS software (version 2021) was used to calculate the statistical power. The statistical power of the study was higher than 0.90 with the eﬀect size of 1.15, α err prob of 0.05, and sample size of 5476. The missing values were filled in by multiple interpolation. All statistical analyses were performed using R software (version 4.4.2). A *P* value < 0.05 was considered to indicate statistical significance.

## Results

### Characteristics of the study participants

A total of 5476 individuals (2684 men and 2792 women) were included in this study, with an average age of 66.92 ± 13.25 years. The baseline characteristics of participants, classified according to RDW tertiles, are shown in Table 1. Compared with the low-RDW population, the high-RDW population was more likely to be older, African American, with CHF, arrhythmias, high level of BMI, BUN, Scr, serum potassium, RDW, WBC, and low glucose levels (Table 1).

**Table 1:** Baseline characteristics of the participants by tertiles of RDW.

Variables	Overall	RDW (%)			*P* value
		Tertile 1 (≤ 14.9)	Tertile 2 (15–16.1)	Tertile 3 (≥ 16.2)	
No. of participants	5476	1758	1868	1850	
Age (years)	66.92 ± 13.25	65.62 ± 14.05	67.27 ± 13.17	67.79 ± 12.43	< 0.001
					< 0.001
< 65	2197 (40.12)	784 (44.60)	729 (39.03)	684 (36.97)	
≥ 65	3279 (59.88)	974 (55.40)	1139 (60.97)	1166 (63.03)	
Sex (n, %)					0.186
Male	2684 (49.01)	831 (47.27)	924 (49.46)	929 (50.22)	
Female	2792 (50.99)	927 (52.73)	944 (50.54)	921 (49.78)	
Ethnicity (n, %)					< 0.001
Caucasian	4108 (75.02)	1377 (78.33)	1387 (74.25)	1344 (72.65)	
African American	642 (11.72)	139 (7.91)	225 (12.05)	278 (15.03)	
Hispanic	304 (5.55)	100 (5.69)	94 (5.03)	110 (5.95)	
Asian	224 (4.09)	78 (4.44)	95 (5.09)	51 (2.76)	
Native American	80 (1.46)	22 (1.25)	23 (1.23)	35 (1.89)	
Other	118 (2.15)	42 (2.39)	44 (2.36)	32 (1.73)	
COPD (n, %)					0.624
No	5077 (92.71)	1629 (92.66)	1740 (93.15)	1708 (92.32)	
Yes	399 (7.29)	129 (7.34)	128 (6.85)	142 (7.68)	
CHF (n, %)					< 0.001
No	4997 (91.25)	1648 (93.74)	1707 (91.38)	1642 (88.76)	
Yes	479 (8.75)	110 (6.26)	161 (8.62)	208 (11.24)	
AMI (n, %)					0.203
No	5281 (96.44)	1684 (95.79)	1808 (96.79)	1789 (96.70)	
Yes	195 (3.56)	74 (4.21)	60 (3.21)	61 (3.30)	
Pneumonia (n, %)					0.657
No	3944 (72.02)	1254 (71.33)	1358 (72.70)	1332 (72.00)	
Yes	1532 (27.98)	504 (28.67)	510 (27.30)	518 (28.00)	
Arrhythmias (n, %)					0.029
No	4772 (87.14)	1549 (88.11)	1642 (87.90)	1581 (85.46)	
Yes	704 (12.86)	209 (11.89)	226 (12.10)	269 (14.54)	
BMI (kg/m^2^)	32.01 ± 9.87	31.29 ± 9.00	32.50 ± 10.46	32.18 ± 10.02	< 0.001
BUN (mg/dL)	35.00 (21.00, 48.00)	28.00 (18.00, 44.00)	38.87 (24.00, 45.00)	36.00 (22.00, 55.00)	< 0.001
Calcium (mg/dL)	8.09 ± 0.80	8.09 ± 0.79	8.08 ± 0.72	8.10 ± 0.89	0.756
Glucose (mg/dL)	167.00 (120.00, 215.00)	173.00 (127.00, 238.00)	184.00 (127.00, 205.00)	150.00 (111.00, 201.00)	< 0.001
Scr (mg/dL)	1.70 (1.03, 2.60)	1.39 (0.92, 2.26)	1.99 (1.13, 2.40)	1.87 (1.10, 3.16)	< 0.001
Serum potassium (mmol/L)	4.18 ± 0.78	4.11 ± 0.75	4.20 ± 0.74	4.24 ± 0.85	< 0.001
Sodium (mmol/L)	138.00 ± 5.88	137.77 ± 5.74	138.14 ± 5.93	138.09 ± 5.92	0.119
Platelets (cells×10^9^/L)	206.01 ± 105.14	204.48 ± 96.41	208.35 ± 95.64	205.10 ± 121.05	0.489
RDW (%)	16.11 ± 2.31	13.92 ± 0.70	15.81 ± 0.38	18.50 ± 2.19	< 0.001
WBC (cells×10^9^/L)	15.19 ± 11.83	14.81 ± 9.06	15.33 ± 10.86	15.42 ± 14.73	0.243

BMI, body mass index; COPD, chronic obstructive pulmonary disease; CHF, congestive heart failure; AMI, acute myocardial infarction; BUN, blood urea nitrogen; RDW, red blood cell distribution width; WBC, white blood cell; Scr, serum creatinine.

### K-M survival analysis curves for 28-day mortality

**Fig 1:**
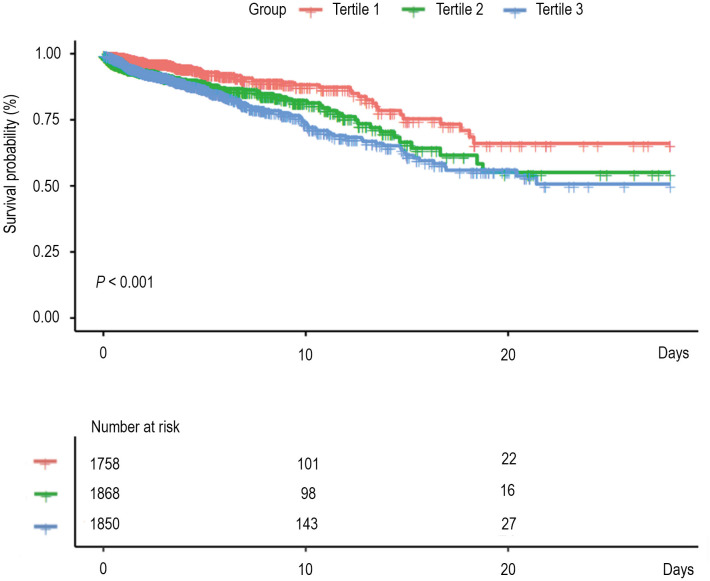
Kaplan-Meier survival curves for 28-day mortality by the RDW tertiles.

Kaplan-Meier survival curves for the probability of 28-day mortality according to RDW tertiles are shown in Fig 1. The results demonstrated that patients in higher RDW tertiles were related to a higher risk of 28-day ICU mortality (log-rank test, *P* < 0.001).

### Univariate and Multivariate logistic regression analysis of the relationship of RDW and 28-day mortality

Table 2 shows the results of the univariate logistic regression analysis. Age, CHF, BMI, BUN, calcium, Scr, serum potassium, platelets, RDW, and WBC count were significantly associated with 28-day mortality. The unadjusted odds ratio (OR) for mortality risk was 1.16 (95% confidence interval [CI]: 1.12, 1.21; *P* < 0.001). Compared with the low tertile of RDW, patients with a high RDW had a stronger correlation (OR = 2.83, 95% CI: 2.16, 3.70; *P* < 0.001). Table 3 shows the results of the multivariate logistic regression analysis. After adjusting for significant covariates in Model 2, RDW was significantly related to mortality. After adjusting other potential confounders in model 3, this correlation remained significant regardless of whether the RDW was considered as a continuous variable (OR = 1.37, 95% CI: 1.26, 1.50; *P* < 0.001) or categorical (OR = 2.44, 95% CI: 1.85, 3.22; *P* < 0.001). This trend was statistically significant (*P* < 0.001).

**Table 2:** Univariate logistic regression analysis of 28-day mortality.

Variable	OR (95% CI)	*P* value
Age (years)		
< 65	Ref.	
≥ 65	1.62 (1.32, 2.00)	< 0.001
Sex (n, %)		
Male	Ref.	
Female	1.03 (0.85, 1.24)	0.801
Ethnicity (n, %)		
Caucasian	Ref.	
African American	0.86 (0.63, 1.19)	0.369
Hispanic	1.36 (0.93, 1.99)	0.109
Asian	0.75 (0.43, 1.30)	0.298
Native American	1.42 (0.70, 2.86)	0.329
Other	1.15 (0.61, 2.16)	0.663
COPD (n, %)		
No	Ref.	
Yes	0.73 (0.48, 1.11)	0.142
CHF (n, %)		
No	Ref.	
Yes	1.41 (1.04, 1.91)	0.028
AMI (n, %)		
No	Ref.	
Yes	1.14 (0.70, 1.87)	0.600
Pneumonia (n, %)		
No	Ref.	
Yes	1.18 (0.96, 1.45)	0.122
Arrhythmias (n, %)		
No	Ref.	
Yes	1.07 (0.81, 1.42)	0.644
BMI (kg/m^2^)		
Tertile 1 (< 27.03)	Ref.	
Tertile 1 (27.03–33.72)	0.86 (0.69, 1.09)	0.216
Tertile 1 (≥ 33.73)	0.79 (0.62, 1.00)	0.048
BUN (mg/dL)	1.01 (1.01, 1.02)	< 0.001
Calcium (mg/dL)	0.76 (0.67, 0.86)	< 0.001
Glucose (mg/dL)	1.00 (1.00, 1.00)	0.248
Scr (mg/dL)	1.11 (1.07, 1.16)	< 0.001
Serum potassium (mmol/L)	1.46 (1.31, 1.63)	< 0.001
Sodium (mmol/L)	1.01 (0.99, 1.03)	0.244
Platelets (cells×10^9^/L)	1.00 (1.00, 1.00)	< 0.001
RDW (%)	1.16 (1.12, 1.21)	< 0.001
RDW (Per SD)	1.42 (1.31, 1.54)	< 0.001
RDW (%)		
Tertile 1(≤ 14.9)	Ref.	
Tertile 2 (15–16.1)	2.06 (1.56, 2.72)	< 0.001
Tertile 3 (≥ 16.2)	2.83 (2.16, 3.70)	< 0.001
WBC (cells×10^9^/L)	1.02 (1.01, 1.02)	< 0.001

BMI, body mass index; COPD, chronic obstructive pulmonary disease; CHF, congestive heart failure; AMI, acute myocardial infarction; BUN, blood urea nitrogen; RDW, red blood cell distribution width; WBC, white blood cells; Scr, serum creatinine; OR, odds ratio; CI, confidence interval; Ref, reference.

**Table 3:** Multivariate logistic regression analysis of the relationship of RDW and 28-day mortality.

RDW (%)	Model 1		Model 2		Model 3	
	OR (95% CI)	*P* value	OR (95% CI)	*P* value	OR (95% CI)	*P* value
Per Unit increase	1.16 (1.12, 1.21)	< 0.001	1.15 (1.11, 1.19)	< 0.001	1.15 (1.10, 1.19)	< 0.001
Per SD increase	1.42 (1.31, 1.54)	< 0.001	1.37 (1.26, 1.50)	< 0.001	1.37 (1.26, 1.50)	< 0.001
Tertile 1 (≤ 14.9)	Ref.		Ref.		Ref.	
Tertile 2 (15–16.1)	2.04 (1.54, 2.70)	< 0.001	1.95 (1.47, 2.59)	< 0.001	1.95 (1.46, 2.59)	< 0.001
Tertile 3 (≥ 16.2)	2.76 (2.11, 3.62)	< 0.001	2.45 (1.86, 3.23)	< 0.001	2.44 (1.85, 3.22)	< 0.001
*P* for trend		< 0.001		< 0.001		< 0.001

Model 1: adjusted for age, sex, and ethnicity; Model 2: adjusted for age, CHF, BMI, BUN, calcium, Scr, serum potassium, platelets, and WBC; Model 3: adjusted for age, sex, and ethnicity, COPD, CHF, AMI, pneumonia, arrhythmias, BMI, BUN, calcium, glucose, Scr, serum potassium, sodium, platelets, and WBC; COPD, chronic obstructive pulmonary disease; CHF, congestive heart failure; AMI, acute myocardial infarction; BMI, body mass index; Scr, serum creatinine; WBC, white blood cells; BUN, blood urea nitrogen; RDW, red cell distribution width; SD, standard deviation; OR, odds ratio; CI, confidence interval; Ref, reference.

### Dose-response relationship between RDW and 28-day mortality

**Fig 2:**
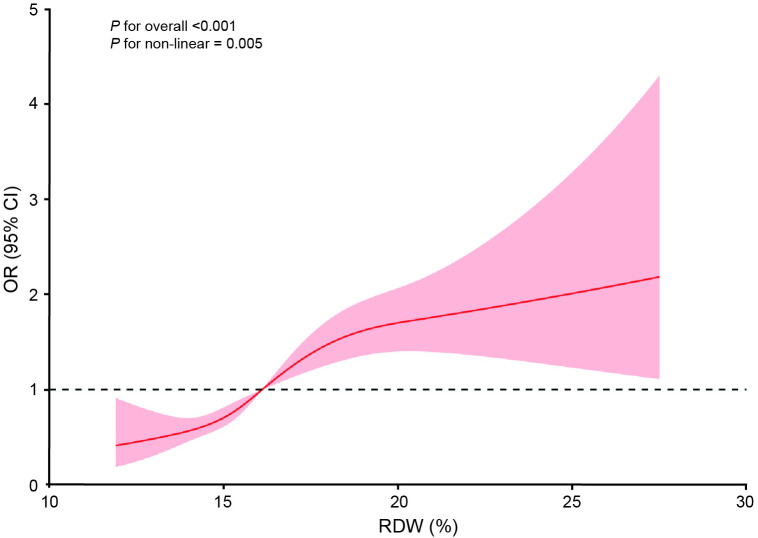
The restricted cubic splines analysis of the dose-response relationship between RDW and 28-day mortality. Red solid lines represent the OR value, and shadows represent the corresponding 95% CI. RDW, red cell distribution width; OR, odds ratio; CI, confidence interval.

Restricted cubic spline (RCS) analysis demonstrated that the risk of ICU mortality increased non-linearly with increasing RDW (*P* for non-linear = 0.005) (Fig 2). In addition, the RDW threshold was 18.95%. On the left of 18.95%, the relationship between RDW and mortality was significant (OR = 1.25, 95% CI: 1.16, 1.35; *P* < 0.001) (Table 4). However, on the left of 18.95%, no significant relationship was found on the left side (OR = 1.04, 95% CI: 0.91, 1.19; *P* = 0.611) (Table 4).

**Table 4:** Threshold effect analysis of the dose-response relationship between RDW and 28-day mortality.

RDW (%)	OR (95% CI)	*P* value
< 18.95	1.25 (1.16, 1.35)	< 0.001
≥ 18.95	1.04 (0.91, 1.19)	0.611

RDW, red cell distribution width; OR, odds ratio; CI, confidence interval.

### Subgroup analysis of the relationship between RDW and 28-day mortality

**Fig 3:**
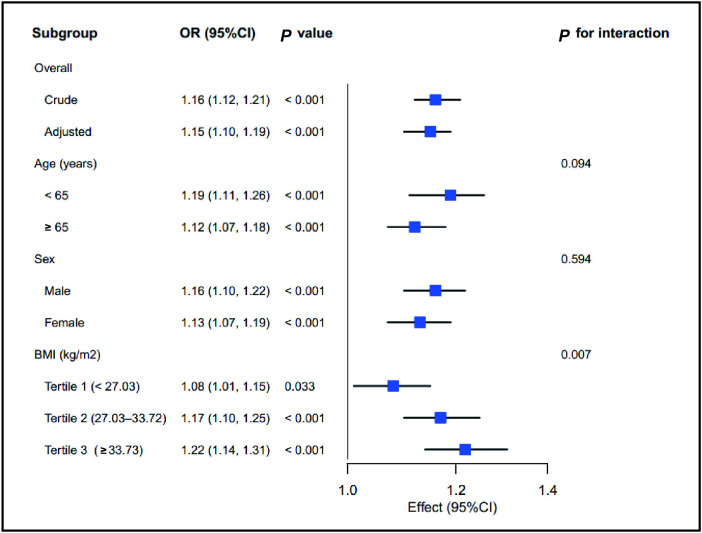
Subgroup analysis of the relationship between RDW and 28-day mortality. BMI, body mass index; OR, odds ratio; CI, confidence interval.

Subgroup analyses were performed to examine the effects of RDW across various variables, as shown in Fig 3. Compared with the lower BMI group, the relationship between RDW and 28-day mortality appeared stronger in the higher BMI group. In addition, a significant interaction effect of BMI on the relationship between RDW and 28-day mortality was observed (*P* = 0.007). Age and sex did not show a significant interaction effect on RDW and 28-day mortality (all *P* > 0.05).

### Mediation analyses of BMI on the correlation between RDW and 28-day mortality

As shown in S2 Fig in S1 File, a mediation analysis was performed to determine the role of BMI in mediating the correlation between RDW and 28-day mortality. The mediation analysis suggested that RDW had a significant direct effect on 28-day mortality (β = 0.022, 95%CI: 0.0156, 0.0274, *P* < 0.001). However, the indirect effect mediated by BMI on the correlation was not significant (β = –0.0001, 95%CI: –0.0003, 0.0001, *P* = 0.380). Therefore, BMI did not play a mediating role in the relationship between RDW and 28-day mortality.

### The incremental predictive value of RDW for 28-day mortality

**Fig 4:**
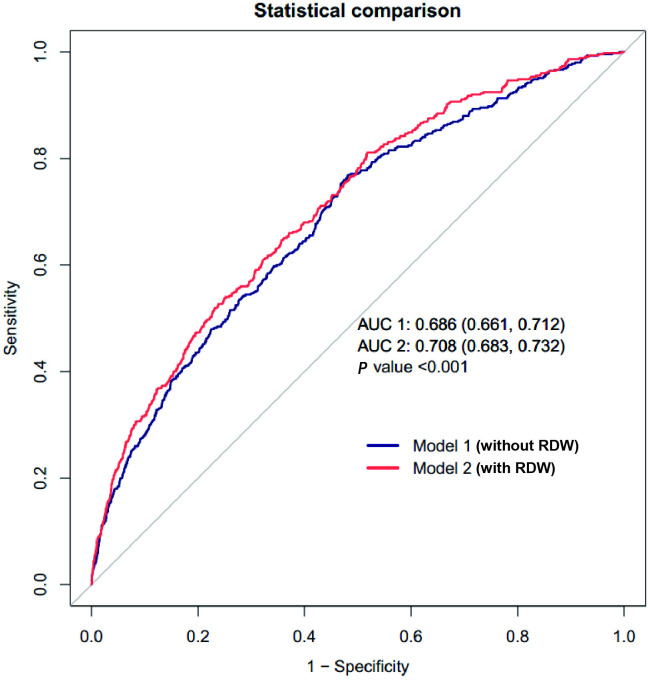
ROC curve analysis of RDW for the prediction of 28-day mortality. ROC, receiver operating characteristic; AUC, area under the curve.

Receiver operating characteristic (ROC) curve analysis was performed to investigate the utility of RDW for mortality prediction. As shown in Fig 4, compared to the area under the curve (AUC) without RDW (AUC 1 = 0.686), the AUC value with RDW (AUC 2 = 0.708) improved the predictive power for 28-day mortality (*P* < 0.001). The SHAP analysis based on the XGBoost model indicated that the contribution of RDW was substantial, further verifying the predictive value of RDW (S3 and S4 Figs in S1 File). In addition, the RDW produced prominent improvements in the NRI and IDI (Table 5).

**Table 5:** The incremental predictive value of RDW for 28-day mortality.

Index	Model 1	Model 2 (95%CI)	*P* value
NRI (Categorical)	Ref.	0.029 (–0.001, 0.059)	0.059
NRI (Continuous)	Ref.	0.284 (0.188, 0.379)	< 0.001
IDI	Ref.	0.012 (0.007, 0.016)	< 0.001

NRI, net reclassification improvement; IDI, integrated discrimination improvement; CI, confidence interval; Ref, reference.

### Sensitivity analyses

To verify the stability of the results, a sensitivity analysis was performed. In the sensitivity analysis, the outcome was 14-day mortality to observe whether the length of the follow-up period impacted the results. The results of the regression analysis, dose relationship, subgroup analysis, mediation analysis, and predictive performance did not change significantly (S5–S11 Figs; S1–S4 Tables in S1 File). We also compared the baseline characteristics of the data before and after interpolation and found no statistical differences, thereby proving that the missing values followed a random pattern (S5 Table in S1 File). Additionally, an E-value was generated to evaluate the potential impact of unmeasured confounders. Unless the OR is higher than 1.81 of an unmeasured confounding factor, the primary findings were considered robust for an unmeasured confounding factor.

## Discussion

To the best of our knowledge, this is the first study to explore the association and predictive value of RDW for ICU mortality in patients with sepsis and diabetes mellitus. After adjusting for confounding factors, RDW showed a significant correlation with mortality. Furthermore, this relationship is non-linear and positive. Moreover, the predictive ability of the baseline model was improved by the inclusion of RDW based on ROC curve analysis and SHAP contribution ranking. A series of sensitivity analyses proved that the results were robust. Of course, our results also need to be interpreted with caution. The ROC curve results show that RDW can increase AUC, but the increase is relatively small. Similarly, the results of NRI and IDI also confirm this point. NRI and IDI have relatively high sensitivity and can effectively identify the improvement ability of variables on the prediction model [23]. The results of NRI and IDI show that the improvement ability of RDW on the prediction model is statistically significant, but the value is relatively small. Therefore, although RDW can enhance the predictive ability of the model, we should not overly exaggerate its individual value. In clinical risk stratification, it would be more appropriate to combine RDW with other indicators for prediction.

Previous studies have revealed a relationship between RDW and mortality in patients with sepsis. A retrospective study of neonatal sepsis revealed that the RDW was higher in non-survivors than in survivors among 500 neonates in Egypt [18]. RDW was also positively correlated with 30-day all-cause mortality [18]. Another prospective study, which enrolled 502 neonates in India, reported that RDW was higher in neonates with sepsis than in controls [19]. Additionally, RDW has been identified as an independent predictor of in-hospital mortality in elderly patients [20]. It is also significantly associated with in-hospital mortality and provides additional prognostic value in patients with sepsis [24,25]. Most of the previous studies were on infants and young children and had relatively small sample sizes. Our research is a large-sample study on adults, which makes up for the deficiencies of previous studies. A meta-analysis that included 11 articles reported the association between RDW and mortality, and it may be a useful predictor of mortality in patients with sepsis [26]. Notably, the heterogeneity of this meta-study was relatively high. Another meta-analysis revealed the superior specificity and sensitivity of RDW in diagnosing mortality in patients with sepsis [27]. A real-world study based on the Medical Information Mart for Intensive Care Database also revealed that RDW has a good predictive value for the short-term mortality of patients with sepsis complicated with diabetes [28]. All these studies support our results.

The possible pathophysiological mechanisms underlying the correlation between RDW and mortality in septic patients are beyond the scope of the present study. We can make inferences and have discussions based on existing literature.

Oxidative stress may decrease the osmotic pressure and cause erythrocyte lysis by activating cation channels on the erythrocyte membrane [29–31]. Multiple inflammatory factors are also closely associated with RDW, such as tumor necrosis factor-α (TNF-α), interleukin-1, and interleukin-6 etc [32]. Inflammatory stress leads to hematopoietic dysfunction in the bone marrow, affecting erythrocyte production and increasing the proportion of reticulocytes in the peripheral blood [32,33]. Inflammatory factors further increase the fragility of reticulocytes, reduce their plasticity, and render them prone to lysis [34]. Inflammation causes the body to release certain substances, leading to the apoptosis of reticulocytes [34]. These processes increase erythrocyte heterogeneity, leading to changes in RDW values.

As is well known, oxidative stress and inflammation are two major characteristics of sepsis and diabetes [35,36]. Therefore, erythrocyte heterogeneity is more evident in patients with sepsis and diabetes mellitus. This may partially explain our results.

Our study had several strengths. First, this study was the first to explore the association and predictive value of RDW for ICU mortality in septic patients with diabetes mellitus. Second, our research is a multi-center study, and the results obtained are more reliable than those from a single center. Third, the size of our sample was relatively large compared to previous studies.

The limitations of this study are as follows. First, some biases could not be avoided, as this was a retrospective cohort study. Second, the relationship between RDW and ICU mortality in septic patients with diabetes mellitus was not associated with causality. Third, the patients included in our study were only from the United States. This may affect the applicability of our results because of differences in populations in other areas.

## Conclusions

In conclusion, RDW was independently associated with ICU mortality in septic patients with diabetes mellitus in a non-linear pattern. Meanwhile, the addition of RDW improved the model’s prediction ability. Taken together, RDW may be a valuable indicator for risk stratification and outcome prediction in septic patients with diabetes mellitus.

## Supporting information

S1 File Supplemental tables, figures, and captions. (DOCX)
