# Supplementary material for: Insights of red cell distribution width for mortality in septic patients with diabetes mellitus: A multicenter cohort study
Source: PLoS One. 2025 Oct 7;20(10):e0333689. doi: 10.1371/journal.pone.0333689 (PMC12503279; doi:10.1371/journal.pone.0333689)
Supplement: S1 File — (DOCX) [file pone.0333689.s001.docx]

**Supplemental tables, figures, and captions**


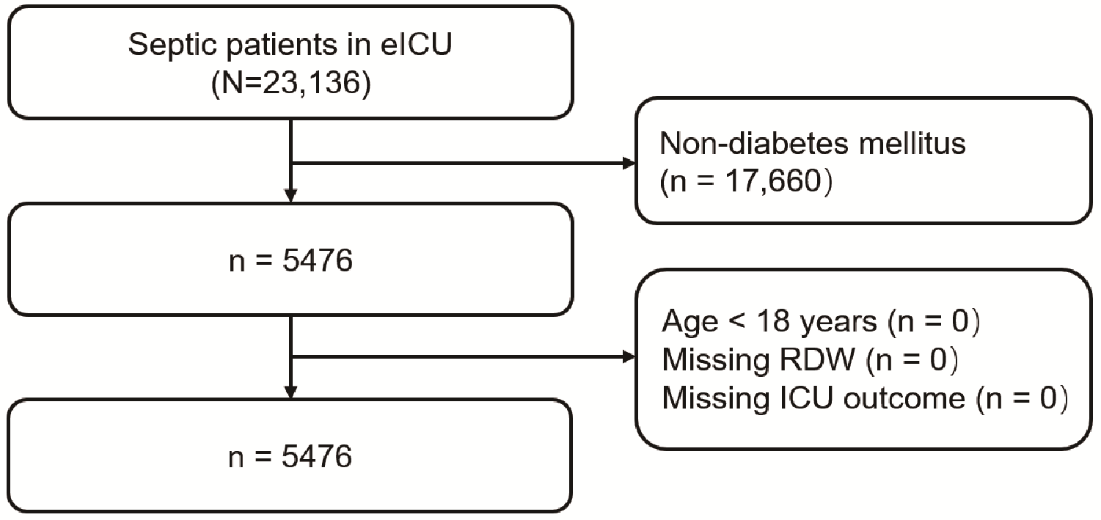


**S1 Fig. Flow chart of screening of population.** RDW, red cell distribution width; ICU, intensive care unit.


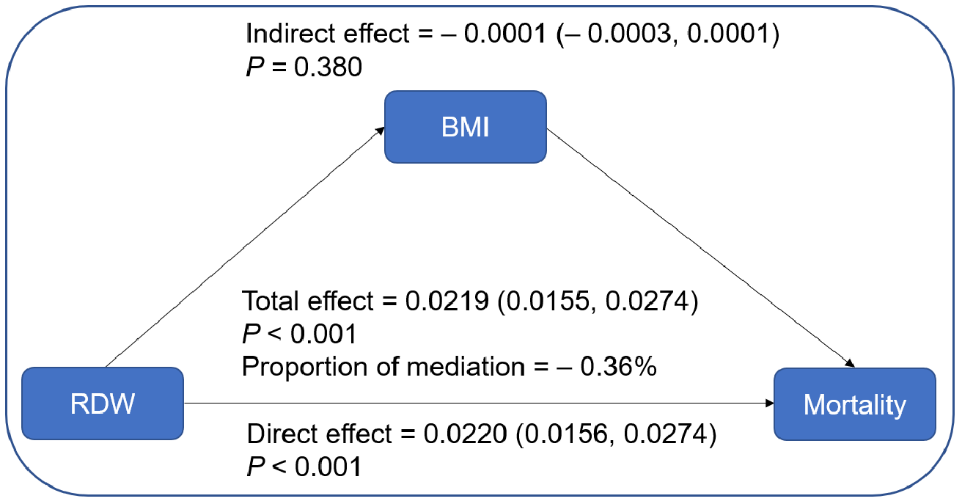


**S2 Fig. Mediation analyses of BMI on the correlation between RDW and 28-day mortality.** RDW, red cell distribution width; BMI, body mass index.


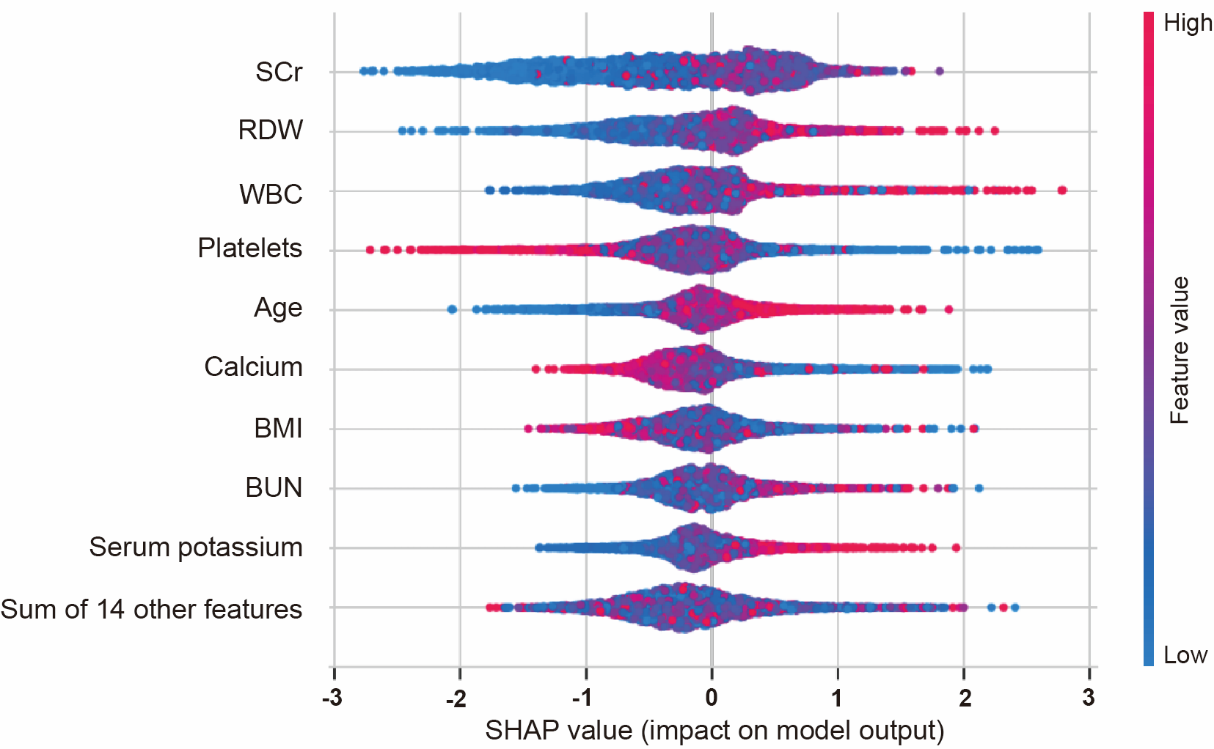


**S3 Fig. SHAP analysis of the tree diagram of XGBoost model characteristics for 28-day mortality.**


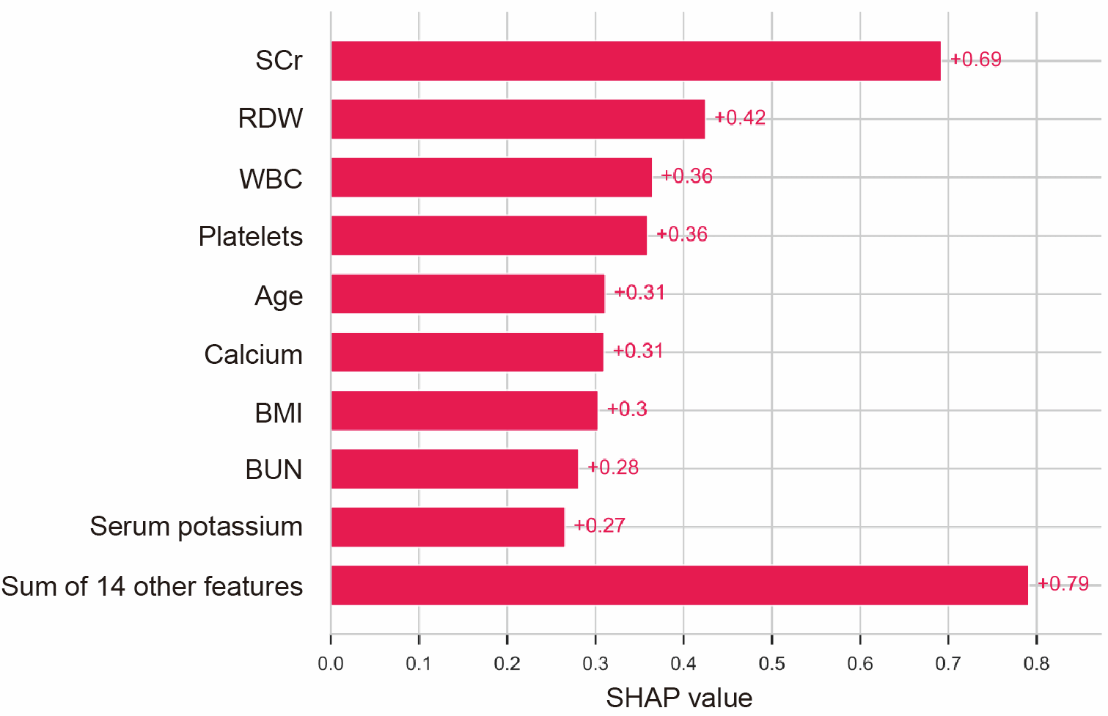


**S4 Fig. SHAP analysis of ranking of importance of XGBoost model features for 28-day mortality.**


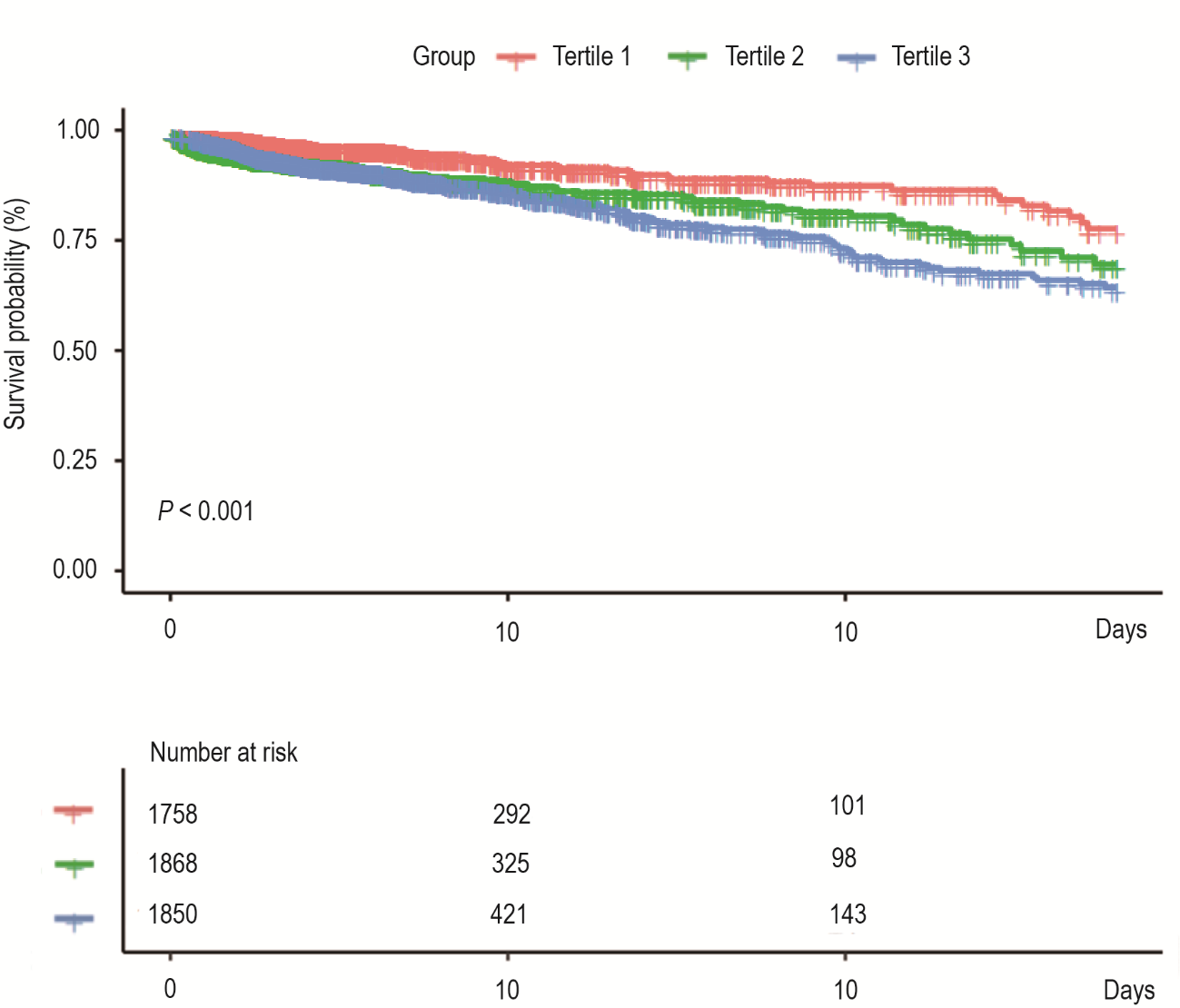


**S5 Fig. Kaplan-Meier survival curves for the incidence of 14-day mortality by the RDW tertiles.**


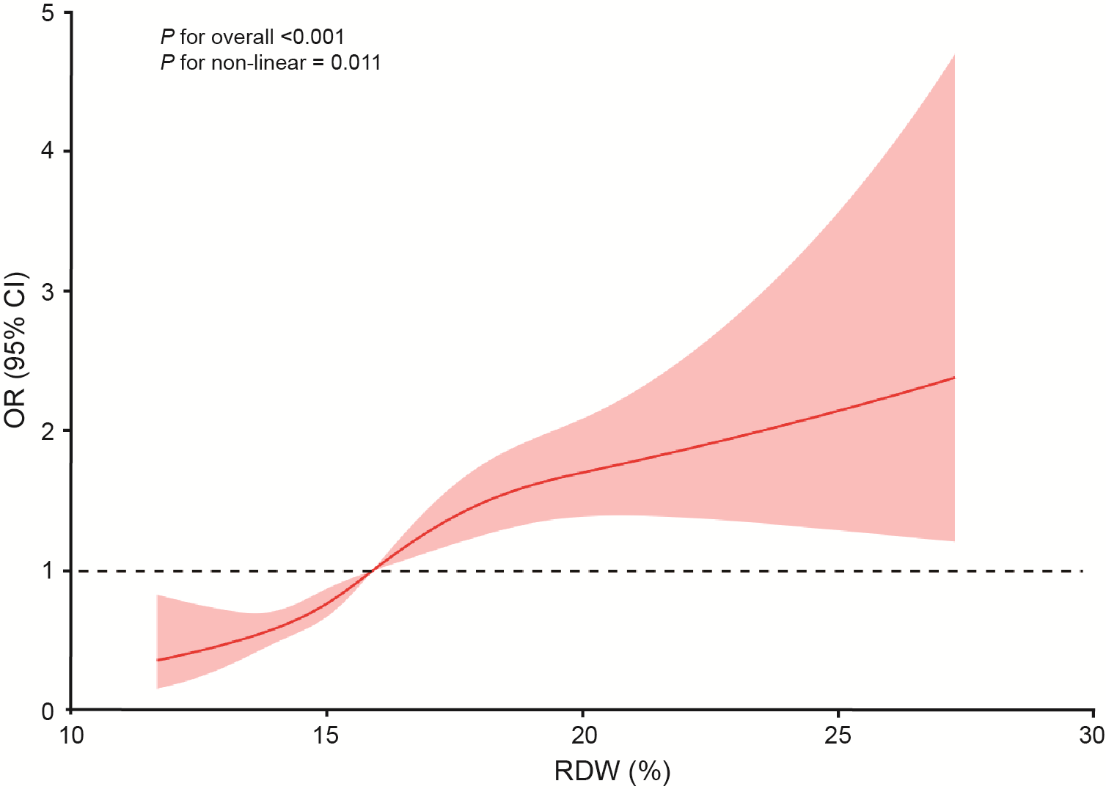


**S6 Fig. The restricted cubic splines analysis of dose-response relationship between RDW and 14-day mortality.** Red solid lines represent the OR value, and shadows represent the corresponding 95% CI. RDW, red cell distribution width; OR, odds rate; CI, confidence interval; Ref., reference.


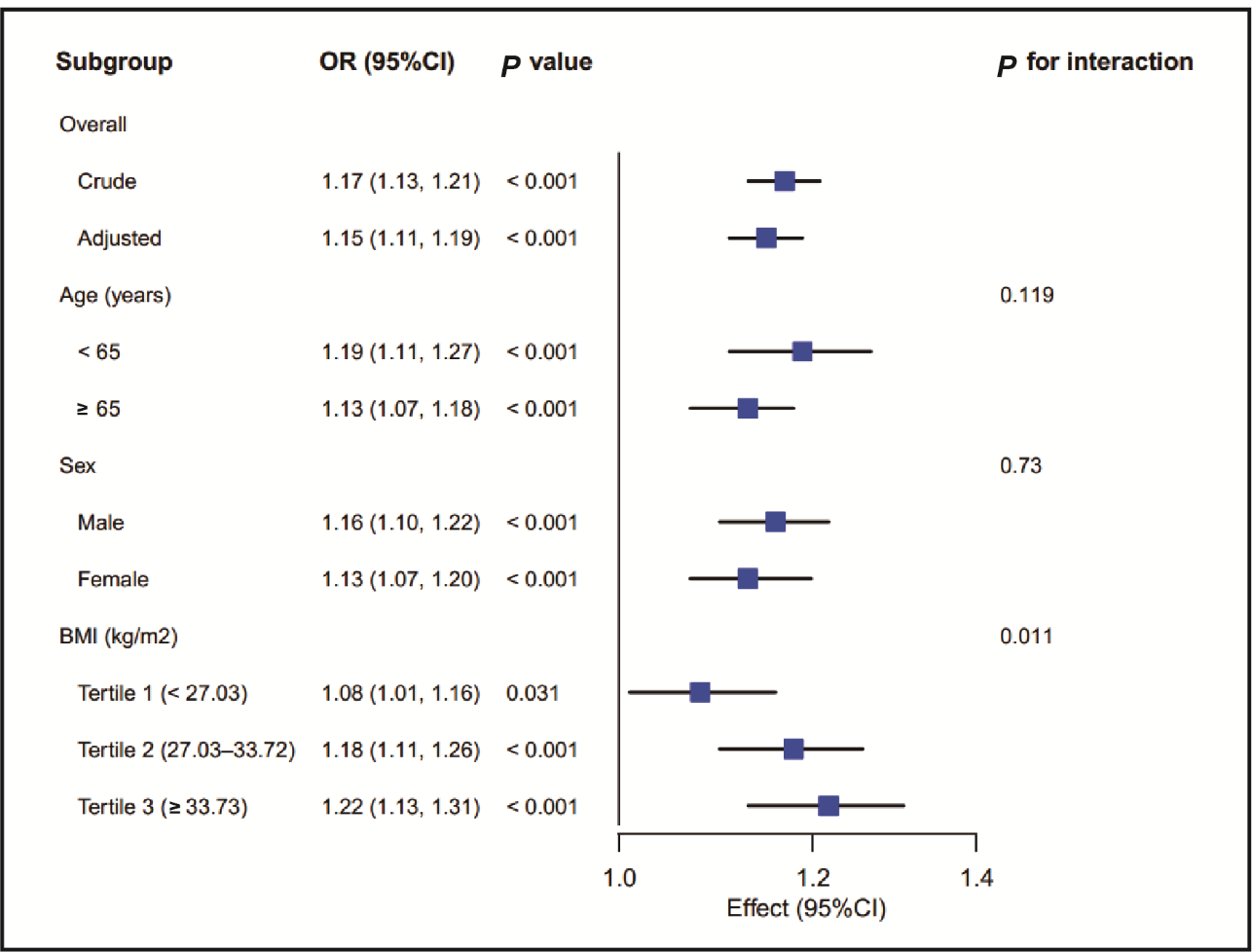


**S7 Fig. Subgroup analysis of relationship between RDW and 14-day mortality.** BMI, body mass index; OR, odds rate; CI, confidence interval.


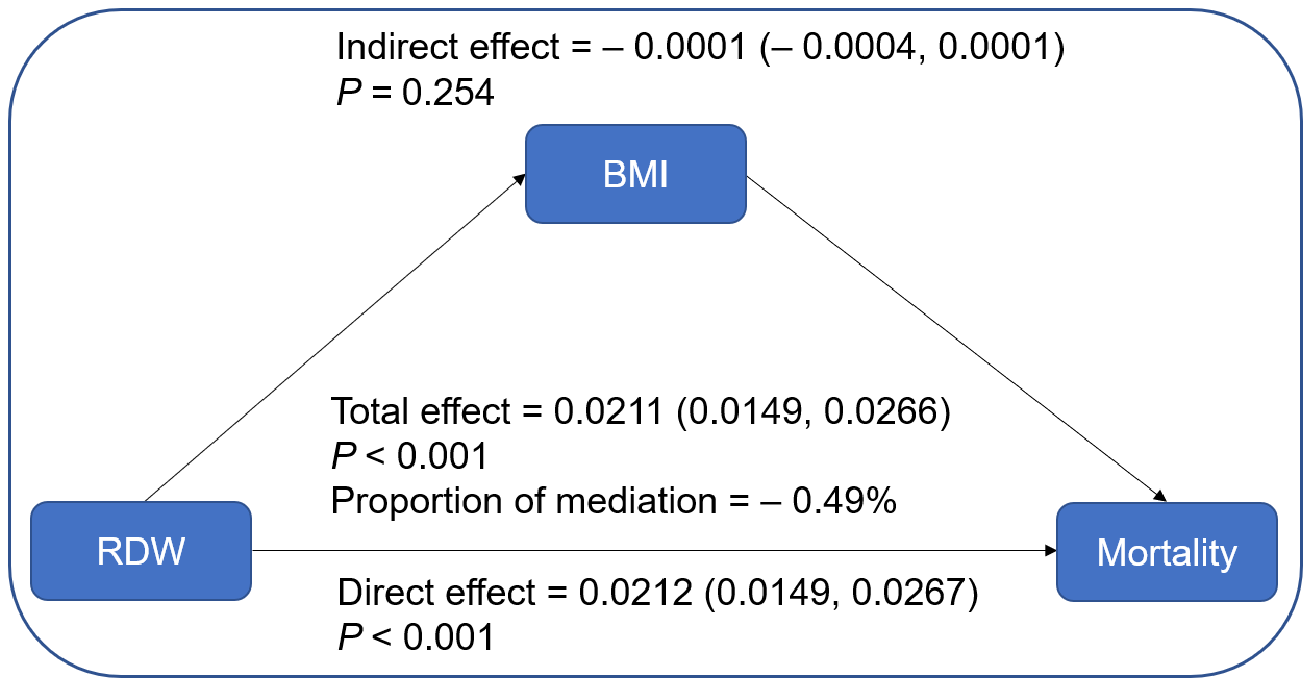


**S8 Fig. Mediation analyses of BMI on the relationship between RDW and 14-day mortality.** RDW, red cell distribution width; BMI, body mass index.


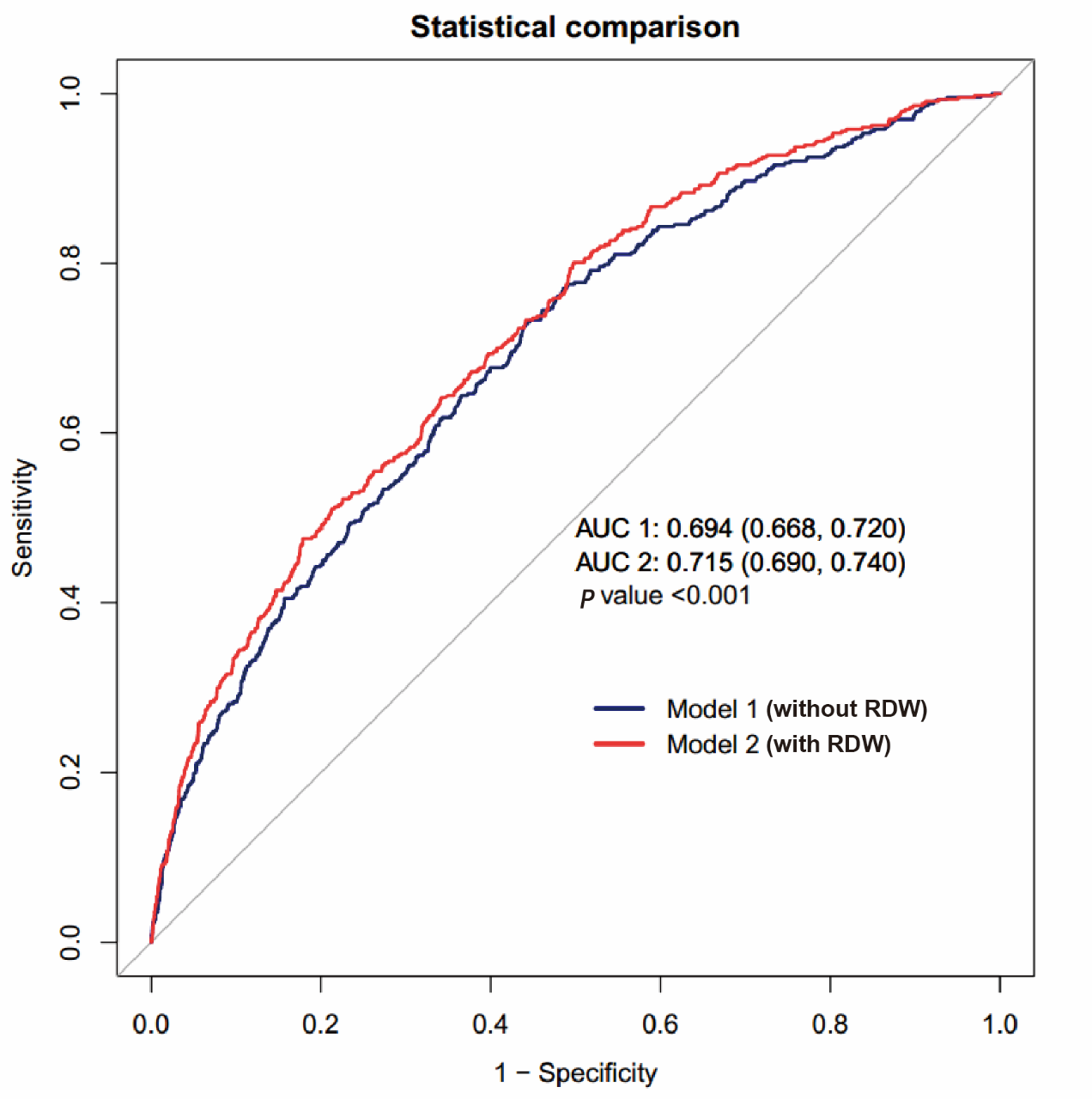


**S9 Fig. ROC curve analysis of RDW for the prediction of 14-day mortality.** ROC, receiver operating characteristic; AUC, area under the curve.


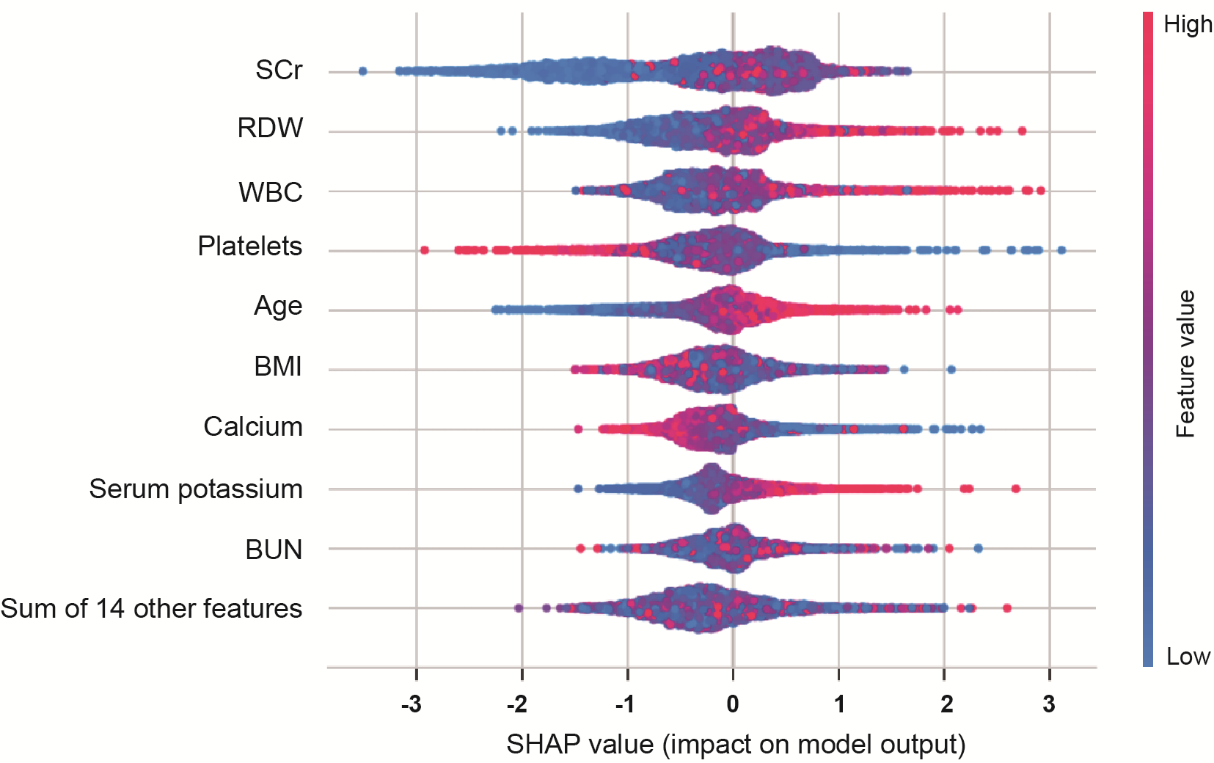


**S10 Fig. SHAP analysis of tree diagram of XGboost model characteristics for 14-day mortality.**


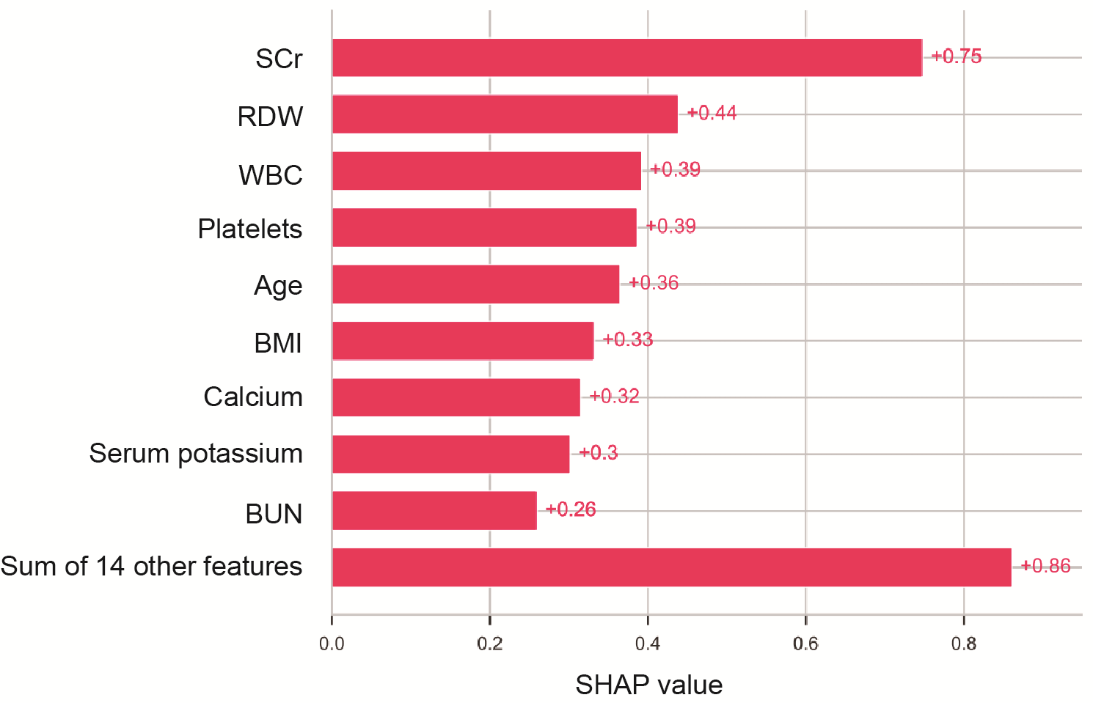


**S11 Fig.** **SHAP analysis of ranking of importance of XGboost model features for 14-day mortality.**

**S1 Table. Univariate logistic regression analysis of 14-day mortality.**

| **Variable** | **OR (95% CI)** | ***P* value** |
| --- | --- | --- |
| Age (years) |  |  |
| < 65 | Ref. |  |
| ≥ 65 | 1.68 (1.36, 2.09) | < 0.001 |
| Sex (n, %) |  |  |
| Male | Ref. |  |
| Female | 1.00 (0.82, 1.22) | 0.977 |
| Ethnicity (n, %) |  |  |
| Caucasian | Ref. |  |
| African American | 0.87 (0.63, 1.21) | 0.419 |
| Hispanic | 1.40 (0.95, 2.05) | 0.087 |
| Asian | 0.73 (0.41, 1.30) | 0.284 |
| Native American | 1.51 (0.75, 3.04) | 0.254 |
| Other | 1.10 (0.57, 2.12) | 0.777 |
| COPD (n, %) |  |  |
| No | Ref. |  |
| Yes | 0.71 (0.46, 1.09) | 0.117 |
| CHF (n, %) |  |  |
| No | Ref. |  |
| Yes | 1.46 (1.08, 1.99) | 0.015 |
| AMI (n, %) |  |  |
| No | Ref. |  |
| Yes | 1.21 (0.74, 1.99) | 0.448 |
| Pneumonia (n, %) |  |  |
| No | Ref. |  |
| Yes | 1.15 (0.93, 1.43) | 0.195 |
| Arrhythmias (n, %) |  |  |
| No | Ref. |  |
| Yes | 1.07 (0.80, 1.43) | 0.640 |
| BMI (kg/m^2^) |  |  |
| Tertile 1 (< 27.03) | Ref. |  |
| Tertile 1 (27.03–33.72) | 0.87 (0.69, 1.10) | 0.255 |
| Tertile 1 (≥ 33.73) | 0.75 (0.59, 0.96) | 0.023 |
| BUN (mg/dL) | 1.01 (1.01, 1.02) | < 0.001 |
| Calcium (mg/dL) | 0.75 (0.66, 0.85) | < 0.001 |
| Glucose (mg/dL) | 1.00 (1.00, 1.00) | 0.293 |
| Scr (mg/dL) | 1.12 (1.07, 1.16) | < 0.001 |
| Serum potassium (mmol/L) | 1.48 (1.32, 1.66) | < 0.001 |
| Sodium (mmol/L) | 1.01 (0.99, 1.02) | 0.403 |
| Platelets (cells×10^9^/L) | 1.00 (1.00, 1.00) | < 0.001 |
| RDW (%) | 1.17 (1.13, 1.21) | < 0.001 |
| RDW (Per SD) | 1.43 (1.31, 1.55) | < 0.001 |
| RDW (%) |  |  |
| Tertile 1 (≤ 14.9) | Ref. |  |
| Tertile 2 (15–16.1) | 2.15 (1.61, 2.87) | < 0.001 |
| Tertile 3 (≥ 16.2) | 2.90 (2.19, 3.83) | < 0.001 |
| WBC (cells×10^9^/L) | 1.02 (1.01, 1.02) | < 0.001 |

COPD, chronic obstructive pulmonary disease; CHF, congestive heart failure; AMI, acute myocardial infarction; BMI, body mass index; BUN, blood urea nitrogen; Scr, serum creatinine; RDW, red cell distribution width; WBC, white blood cells.

**S2 Table. Multivariate logistic regression analysis of the relationship of RDW and 14-day mortality.**

| **RDW (%)** | **Model 1** |  | **Model 2** |  | **Model 3** | |  | |
| --- | --- | --- | --- | --- | --- | --- | --- | --- |
|  | **OR (95% CI)** | ***P* value** | **OR (95% CI)** | ***P* value** | **OR (95% CI)** | | ***P* value** | |
| Per Unit increase | 1.17 (1.12, 1.21) | < 0.001 | 1.15 (1.11, 1.19) | < 0.001 | | 1.15 (1.11, 1.19) | | < 0.001 |
| Per SD increase | 1.42 (1.31, 1.55) | < 0.001 | 1.38 (1.27, 1.51) | < 0.001 | | 1.38 (1.26, 1.51) | | < 0.001 |
| Tertile 1 (≤ 14.9) | Ref. |  | Ref. |  | | Ref. | |  |
| Tertile 2 (15–16.1) | 2.12 (1.59, 2.84) | < 0.001 | 2.04 (1.52, 2.74) | < 0.001 | | 2.04 (1.52, 2.74) | | < 0.001 |
| Tertile 3 (≥ 16.2) | 2.82 (2.13, 3.73) | < 0.001 | 2.50 (1.88, 3.33) | < 0.001 | | 2.49 (1.87, 3.33) | | < 0.001 |
| *P* for trend |  | < 0.001 |  | < 0.001 | |  | | < 0.001 |

Model 1: adjusted for age, sex, and ethnicity; Model 2: adjusted for age, CHF, BMI, BUN, calcium, Scr, serum potassium, platelets, WBC; Model 3: adjusted for age, sex, and ethnicity, COPD, CHF, AMI, pneumonia, arrhythmias, BMI, BUN, calcium, glucose, Scr, serum potassium, sodium, platelets, and WBC; COPD, chronic obstructive pulmonary disease; CHF, congestive heart failure; AMI, acute myocardial infarction; BMI, body mass index; Scr, serum creatinine; WBC, white blood cells; BUN, blood urea nitrogen; RDW, red cell distribution width; OR, odds rate; CI, confidence interval; Ref., reference.

**S3 Table. Threshold effect analysis of dose-response relationship between RDW and 14-day mortality.**

| **RDW (%)** | **OR (95% CI)** | ***P* value** |
| --- | --- | --- |
| < 18.31 | 1.27 (1.16, 1.39) | < 0.001 |
| ≥ 18.31 | 1.08 (0.96, 1.22) | 0.179 |

RDW, red cell distribution width; OR, odds rate; CI, confidence interval.

**S4 Table. The incremental predictive value of RDW for 14-day mortality.**

| **Index** | **Model 1** | **Model 2 (95%CI)** | ***P* value** |
| --- | --- | --- | --- |
| NRI(Categorical) | Ref. | 0.051 (0.019, 0.083) | 0.002 |
| NRI(Continuous) | Ref. | 0.271 (0.173, 0.368) | < 0.001 |
| IDI | Ref. | 0.012 (0.007, 0.016) | < 0.001 |

NRI, net reclassification improvement; IDI, integrated discrimination improvement; CI, confidence interval; Ref., reference.

**S5 Table. Comparison of baseline characteristics before and after interpolation.**

| **Variables** | **Missing data**  **(n, %)** | **Before multiple**  **imputation** | **After multiple**  **imputation** | ***P* value** |
| --- | --- | --- | --- | --- |
| Age (years) | 0 |  |  |  |
| Sex (n, %) | 0 |  |  |  |
| Ethnicity (n, %) | 21 (0.38) |  |  | 1.000 |
| Caucasian |  | 4087 (74.92) | 4108 (75.02) |  |
| African American |  | 642 (11.77) | 642 (11.72) |  |
| Hispanic |  | 304 (5.57) | 304 (5.55) |  |
| Asian |  | 224 (4.11) | 224 (4.09) |  |
| Native American |  | 80 (1.47) | 80 (1.46) |  |
| Other |  | 118 (2.16) | 118 (2.15) |  |
| COPD (n, %) | 0 |  |  |  |
| CHF (n, %) | 0 |  |  |  |
| AMI (n, %) | 0 |  |  |  |
| Pneumonia (n, %) | 0 |  |  |  |
| Arrhythmias (n, %) | 0 |  |  |  |
| BMI (kg/m^2^) | 192 (3.51) | 32.01 ± 10.05 | 32.01 ± 9.87 | 1.000 |
| BUN (mg/dL) | 769 (14.04) | 32.00 (20.00, 50.00) | 35.00 (21.00, 48.00) | 0.186 |
| Calcium (mg/dL) | 445 (8.13) | 8.09 ± 0.84 | 8.09 ± 0.80 | 1.000 |
| Glucose (mg/dL) | 427 (7.80) | 165.00 (117.00, 212.00) | 167.00 (120.00, 215.00) | 0.175 |
| Scr (mg/dL) | 387 (7.07) | 1.68 (1.02, 2.61) | 1.70 (1.03, 2.60) | 0.138 |
| Serum potassium (mmol/L) | 372 (6.79) | 4.18 ± 0.81 | 4.18 ± 0.78 | 0.999 |
| Sodium (mmol/L) | 379 (6.92) | 138.00 ± 6.08 | 138.00 ± 5.88 | 0.471 |
| Platelets (cells×10^9^/L) | 568 (10.37) | 206.01 ± 111.05 | 206.01 ± 105.14 | 0.195 |
| RDW (%) | 0 |  |  |  |
| WBC (cells×10^9^/L) | 536 (9.79) | 15.19 ± 11.65 | 15.19 ± 11.83 | 0.273 |

COPD, chronic obstructive pulmonary disease; CHF, congestive heart failure; AMI, acute myocardial infarction; BMI, body mass index; Scr, serum creatinine; WBC, white blood cells; BUN, blood urea nitrogen; RDW, red cell distribution width.
